# Supplementary figures and images for: Fusion with heat-resistant obscure (Hero) proteins have the potential to improve the molecular property of recombinant proteins
Source: PLoS One. 2022 Jun 17;17(6):e0270097. doi: 10.1371/journal.pone.0270097 (PMC9205492; doi:10.1371/journal.pone.0270097)

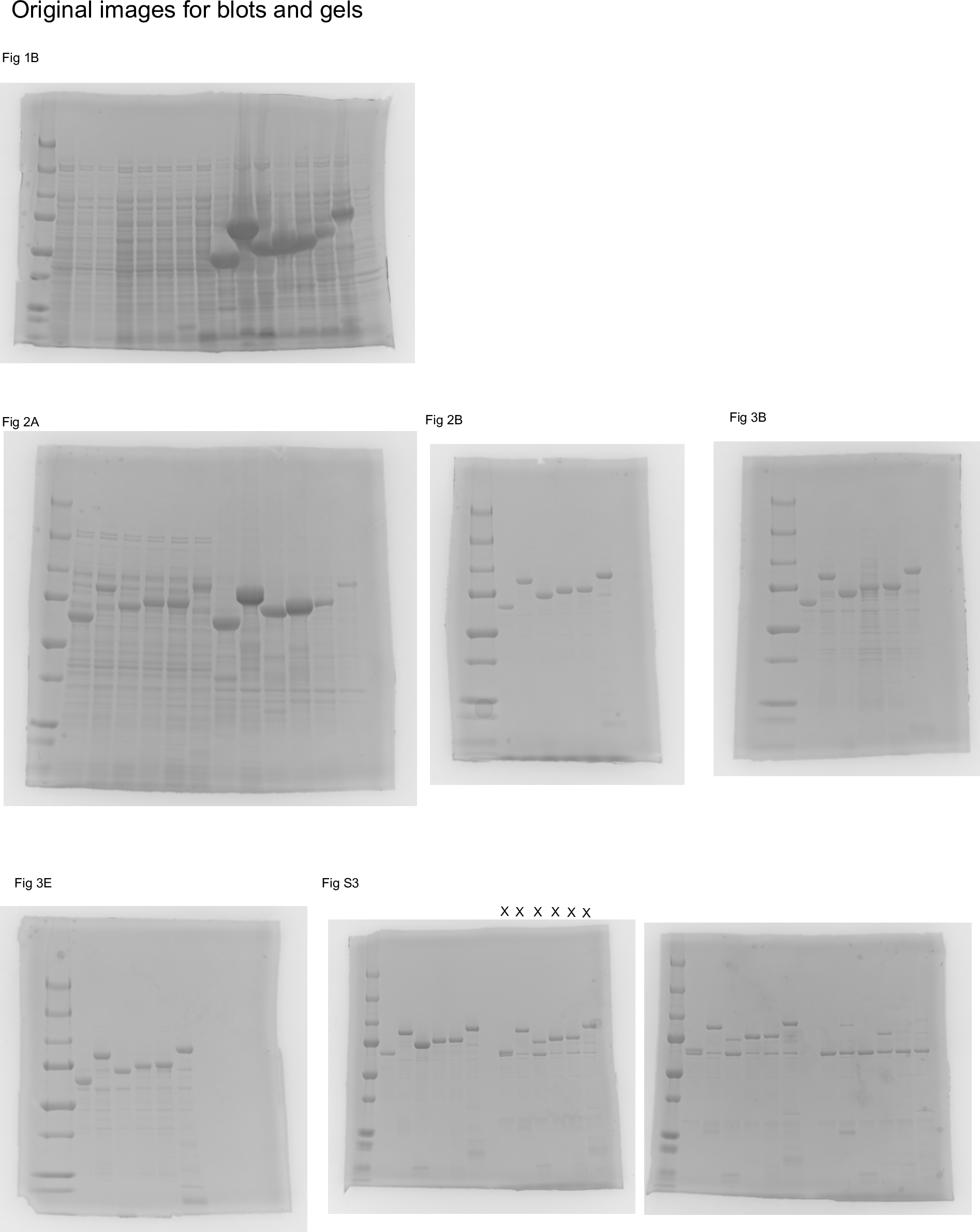

Supplement: S1 Raw images — (TIF) [file pone.0270097.s001.tif]

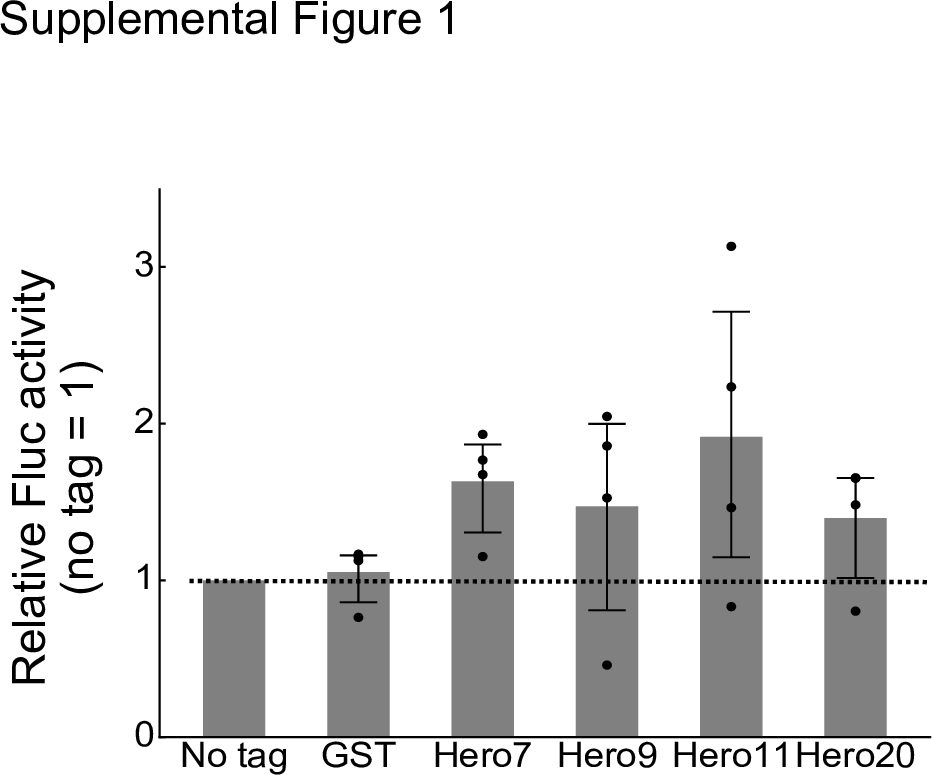

Supplement: S1 Fig — Basal Fluc activities before the stress tests in Fig 2. P-values were calculated by the Steel-Dwass test against no tag. None showed p < 0.05. (TIF) [file pone.0270097.s003.tif]

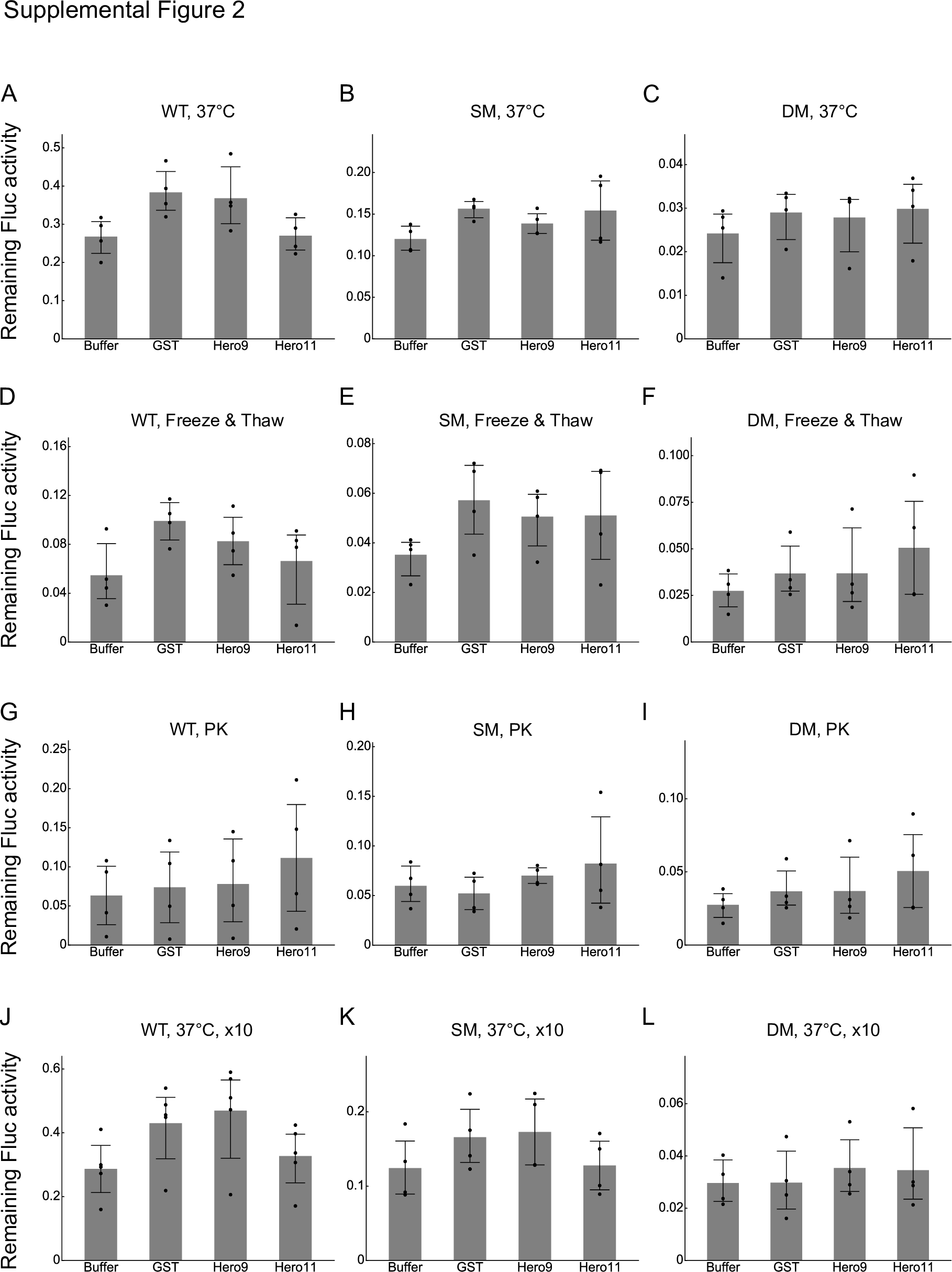

Supplement: S2 Fig — (A). Heat treatment of Fluc-WT mixed with buffer alone, equimolar GST, Hero9 or Hero11 at 37°C for 20 min. (B). Heat treatment of Fluc-SM mixed with buffer alone, equimolar GST, Hero9 or Hero11 at 37°C for 20 min. (C). Heat treatment of Fluc-DM mixed with buffer alone, equimolar GST, Hero9 or Hero11 at 37°C for 10 min. (D)Freeze-thaw cycles of Fluc-WT mixed with buffer alone, equimolar GST, Hero9 or Hero11. (E)Freeze-thaw cycles of Fluc-SM mixed with buffer alone, equimolar GST, Hero9 or Hero11. (F)Freeze-thaw cycles of Fluc-DM mixed with buffer alone, equimolar GST, Hero9 or Hero11. (G)PK treatment of Fluc-WT mixed with buffer alone, equimolar GST, Hero9 or Hero11 for 30 min. (H)PK treatment of Fluc-SM mixed with buffer alone, equimolar GST, Hero9 or Hero11 for 30 min. (I)PK treatment of Fluc-DM mixed with buffer alone, equimolar GST, Hero9 or Hero11 for 30 min. (J)Heat treatment of Fluc-WT mixed with buffer alone, 10-fold GST, Hero9 or Hero11 at 37°C for 20 min. (K)Heat treatment of Fluc-SM mixed with buffer alone, 10-fold GST, Hero9 or Hero11 at 37°C for 20 min. (L)Heat treatment of Fluc-DM mixed with buffer alone, 10-fold GST, Hero9 or Hero11 at 37°C for 20 min. For all the data, p-values were calculated by Tukey HSD against no tag. None showed p < 0.05. (TIF) [file pone.0270097.s004.tif]

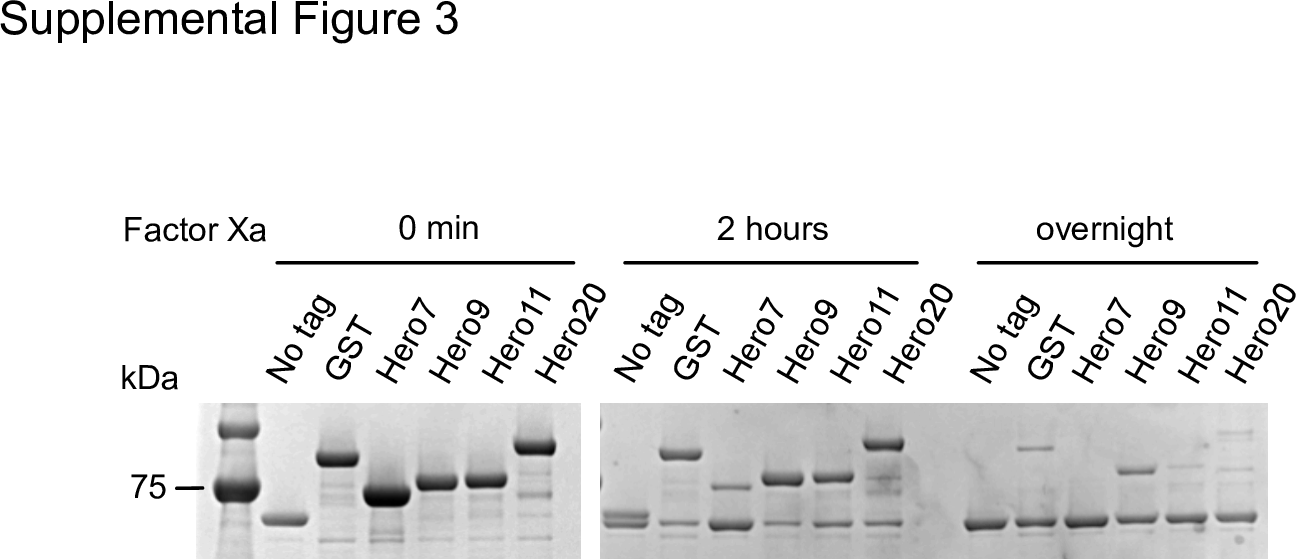

Supplement: S3 Fig — (TIF) [file pone.0270097.s005.tif]
